# Lobular Breast Carcinoma Revealed by a Stenotic Ileal Metastasis

**DOI:** 10.7759/cureus.93473

**Published:** 2025-09-29

**Authors:** Armel Luvumbu, Denis Tack, Jean-Christophe Lebrun

**Affiliations:** 1 Emergency Medicine, Centre Hospitalier EpiCURA - Site de Ath, Ath, BEL; 2 Radiology, Centre Hospitalier EpiCURA - Site de Ath, Ath, BEL

**Keywords:** abdominal surgical emergency, computed tomography imaging, emergency department, emergency medicine, imaging, invasive lobular carcinoma, metastasis, occult carcinoma, small bowel obstruction

## Abstract

Small bowel obstruction (SBO) is a condition frequently encountered in the emergency department. We describe a case of a 49-year-old woman admitted for acute abdominal pain due to an ileal stenosis that proved to be metastatic invasive lobular carcinoma of the breast. Mammography and ultrasonography were negative, while breast MRI showed only subtle lesions. Histology combined with immunohistochemistry confirmed the diagnosis, consistent with an occult primary tumor. The patient underwent segmental ileal resection, followed by hormonal therapy combined with a CDK4/6 inhibitor, with an uncomplicated early postoperative course. This report underlines the importance of considering atypical causes of acute SBO in women, particularly when initial imaging is inconclusive.

## Introduction

Small bowel obstruction (SBO) is a potentially life-threatening condition commonly encountered in the emergency department. In a systematic review on the management and treatment of SBO, neoplasms occurred in 5-10% of cases, with the majority of SBO caused by adhesive obstructions (60-70%), and a minority by inflammatory bowel diseases (IBDs) such as Crohn’s disease (5-7%) [1]. Among neoplasms, breast cancer, one of the most commonly diagnosed cancers in the world, with an estimated 2.26 million cases recorded in 2020, and the leading cause of cancer mortality among females [2], rarely metastasizes to the digestive tract. Gastrointestinal (GI) metastases of breast cancer are more frequent in invasive lobular carcinoma, with an incidence ranging from 4% to 18% [3,4]. Acute intestinal obstruction as an initial presentation remains rare and diagnostically challenging, especially in emergency settings. We report a case of tumoral stenosis of the ileum revealing an occult lobular breast carcinoma.

## Case presentation

A 49-year-old woman presented to the emergency department with acute and intense abdominal pain. The pain had been increasing for two weeks, peaking on the day of presentation. She reported multiple episodes of abdominal pain and diarrhea over the past two months, and on one occasion, this was accompanied by vomiting.

It had led to two prior visits to the emergency department. During one of these visits, one month earlier, a computed tomography abdominal scan showed an ileal lesion suggestive of ileitis, compatible with an IBD. A gastroenterological follow-up was planned.

On this third visit, the patient's vital signs were normal, and she was afebrile. Her medical history included a hiatal hernia, hypothyroidism, gastroesophageal reflux disease, and a hearing loss managed with a hearing aid. She reported no smoking history and moderate alcohol consumption. Abdominal palpation revealed tenderness in both iliac fossae and the hypogastrium. Laboratory tests were unremarkable, besides an elevated white blood count, and showed no significant inflammatory syndrome (Table 1).

**Table 1:** Laboratory results. Besides an elevated white blood cell count, the results were unremarkable. MCV: mean corpuscular volume; MCH: mean corpuscular hemoglobin; MCHC: mean corpuscular hemoglobin concentration; CRP: C-reactive protein; eGFR: estimated glomerular filtration rate; MDRD: Modification of Diet in Renal Disease; CPK: creatine phosphokinase; LDH: lactate dehydrogenase; ALT: alanine aminotransferase; GPT: glutamic pyruvic transaminase; AST: aspartate aminotransferase; GOT: glutamic oxaloacetic transaminase; GGT: gamma-glutamyl transferase; TSH: thyroid-stimulating hormone.

Parameter	Result	Reference range	Interpretation
White blood cells	12.15 x10^3/mm³	3.9 – 9.5	High
Red blood cells	4.97 x10^6/mm³	4.0 – 5.2	Normal
Hematocrit	45.7%	36 – 46	Normal
Hemoglobin	14.8 g/dL	12.0 – 16.0	Normal
MCV	92.0 fL	79 – 99	Normal
MCH	29.8 pg	26 – 34	Normal
MCHC	32.4 g/dL	31 – 37	Normal
Platelets	352 x10^3/mm³	150 – 400	Normal
Mean platelet volume	11.0 fL	6 – 13	Normal
Anisocytosis index	14%	0 – 16	Normal
Neutrophils (relative)	74.3%	40 – 70	High
Lymphocytes (relative)	19.1%	20 – 40	Low
Monocytes (relative)	4.9%	0.1 – 10	Normal
Eosinophils (relative)	1.5%	<6.0	Normal
Basophils (relative)	0.2%	0 – 1	Normal
Neutrophils (absolute)	9.03 x10^3/mm³	1.8 – 7.0	High
Lymphocytes (absolute)	2.32 x10^3/mm³	1.5 – 4.0	Normal
Monocytes (absolute)	0.60 x10^3/mm³	0.1 – 1.0	Normal
Eosinophils (absolute)	0.18 x10^3/mm³	0 – 0.50	Normal
Basophils (absolute)	0.03 x10^3/mm³	0.00 – 0.05	Normal
CRP	8.31 mg/L	<10.0	Normal
Ferritin	21.0 µg/L	30 – 400	Low
Urea	37 mg/dL	15 – 36	High
Creatinine	0.72 mg/dL	0.52 – 1.04	Normal
eGFR (MDRD)	86.1 mL/min/1.73m²	>60	Normal
Uric acid	4.99 mg/dL	2.5 – 6.2	Normal
Total proteins	77.3 g/L	63.0 – 82.0	Normal
Albumin	45.4 g/L	35.0 – 50.0	Normal
Bilirubin (total)	0.50 mg/dL	0.2 – 1.3	Normal
Glucose	97 mg/dL	65 – 100	Normal
Sodium	137 mmol/L	137 – 145	Normal
Potassium	4.23 mmol/L	3.50 – 5.10	Normal
Chloride	100 mmol/L	98 – 107	Normal
Bicarbonate	24.7 mmol/L	22.0 – 30.0	Normal
Calcium	2.44 mmol/L	2.10 – 2.55	Normal
Phosphorus	1.41 mmol/L	0.81 – 1.45	Normal
Magnesium	0.86 mmol/L	0.66 – 1.07	Normal
CPK	112 U/L	30 – 135	Normal
LDH	258 U/L	120 – 246	High
Lipase	90 U/L	23 – 300	Normal
Alkaline phosphatase	64 U/L	38 – 126	Normal
ALT (GPT)	43 U/L	<35	High
AST (GOT)	43 U/L	14 – 36	High
GGT	19 U/L	12 – 43	Normal
TSH	1.27 mU/L	0.40 – 4.20	Normal

**Figure 1:**
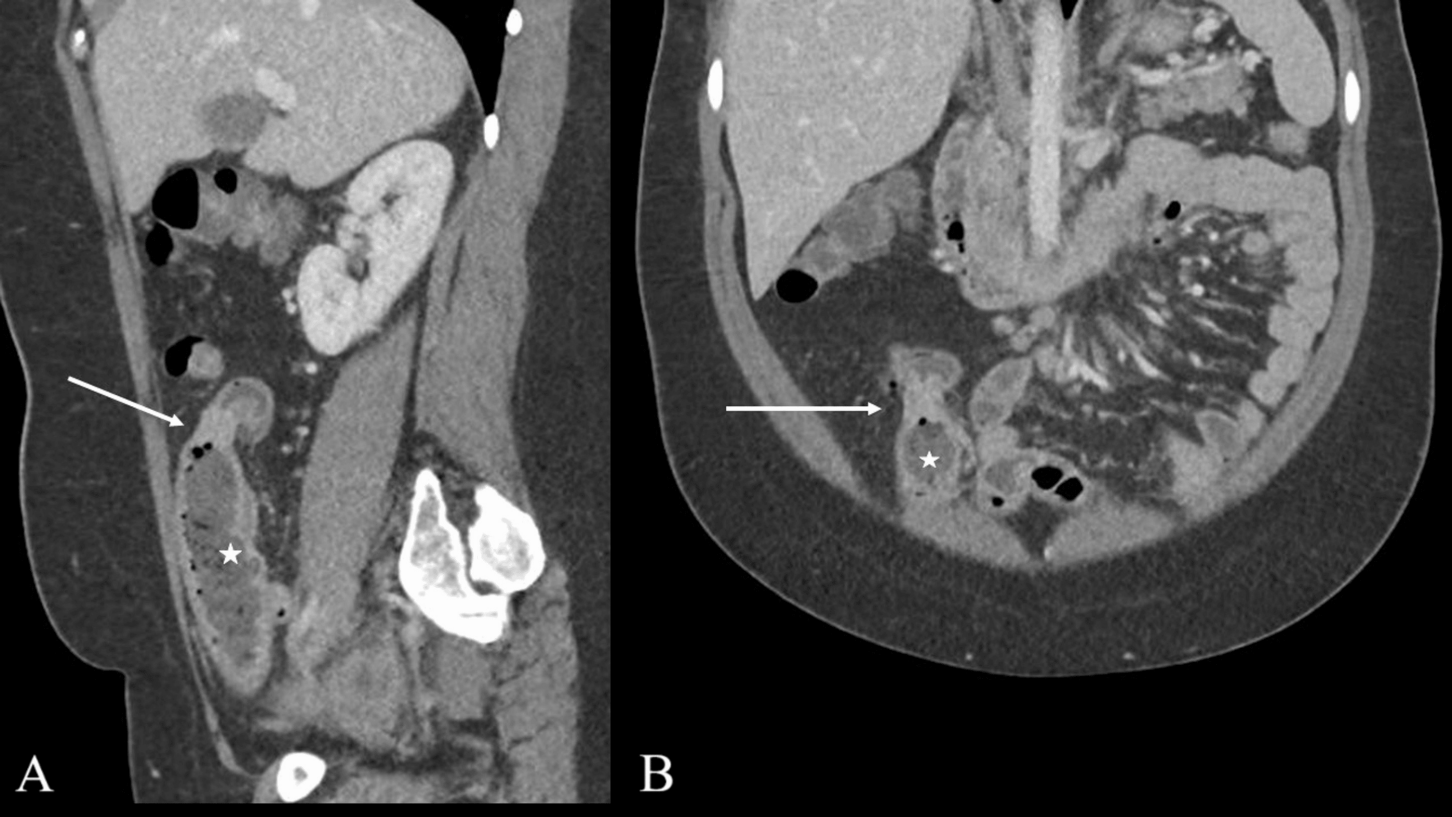
Abdominal tomodensitometry. Sagittal (A) and coronal (B) views of the abdominal CT after intravenous contrast administration showing a circumferential stenotic ileal wall lesion (arrows) with upstream small bowel dilation and a feces sign, indicating delayed transit.

An emergency contrast-enhanced abdominal CT scan revealed a circumferential stenosis of the ileum with upstream dilation (Figure 1).

The patient was hospitalized, and conservative treatment with nasogastric decompression was started.

**Figure 2:**
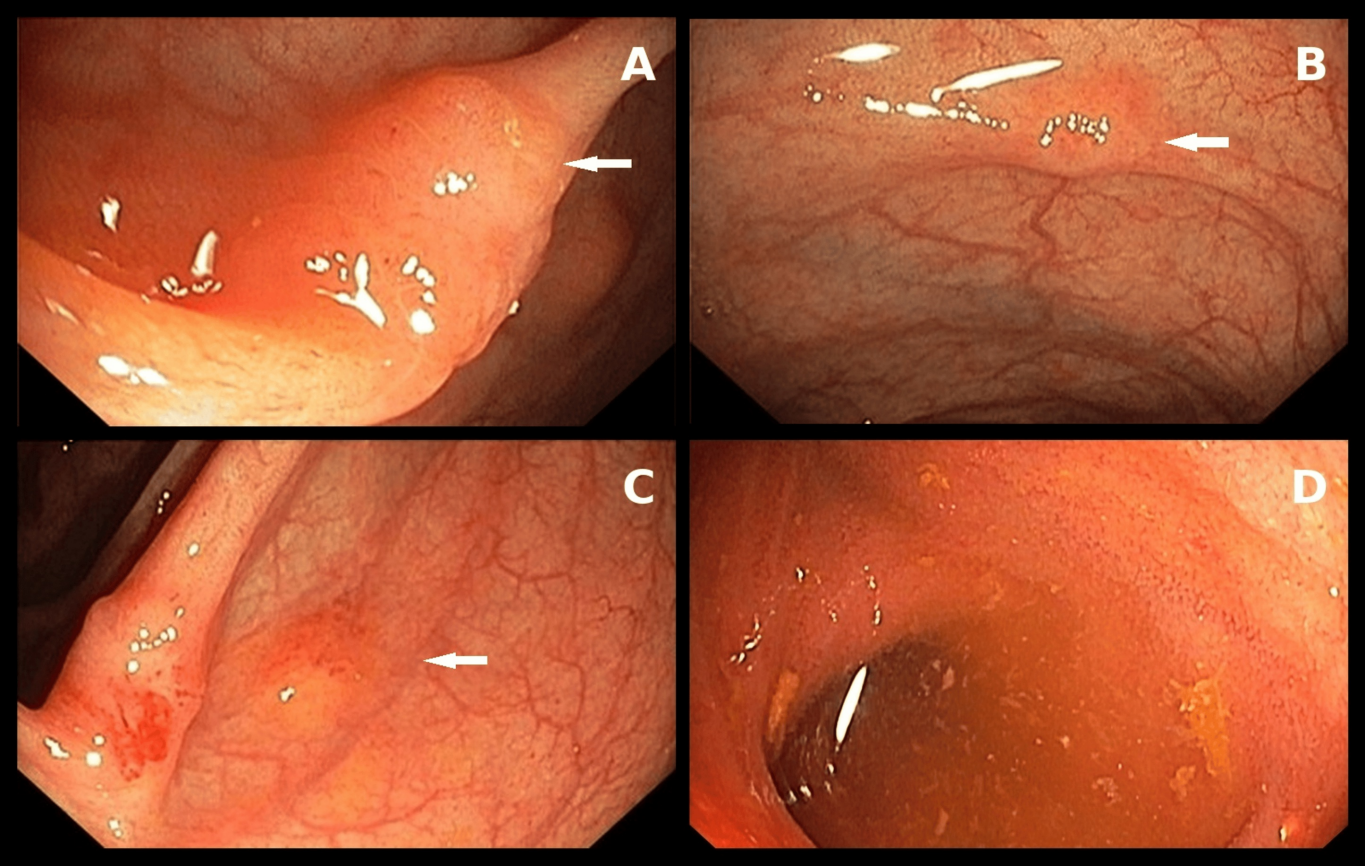
Colonoscopy. Pseudopolypoid lesions (arrows) in the right colon (A-C), and normal ileum (D).

A colonoscopy was performed. The procedure revealed small pseudopolypoid lesions measuring approximately 5-6 mm, predominantly in the caecum and right colon (Figure 2). Biopsies were obtained; on forceps contact, the tissue was described as unusually firm. The terminal ileum was explored for 10 cm beyond the valve, appearing normal, as the stenotic segment could not be reached.

The patient’s condition worsened during her hospitalization, with vomiting and cessation of bowel movements, consistent with SBO. She subsequently underwent a segmental small bowel resection with a side-to-side anastomosis.

**Figure 3:**
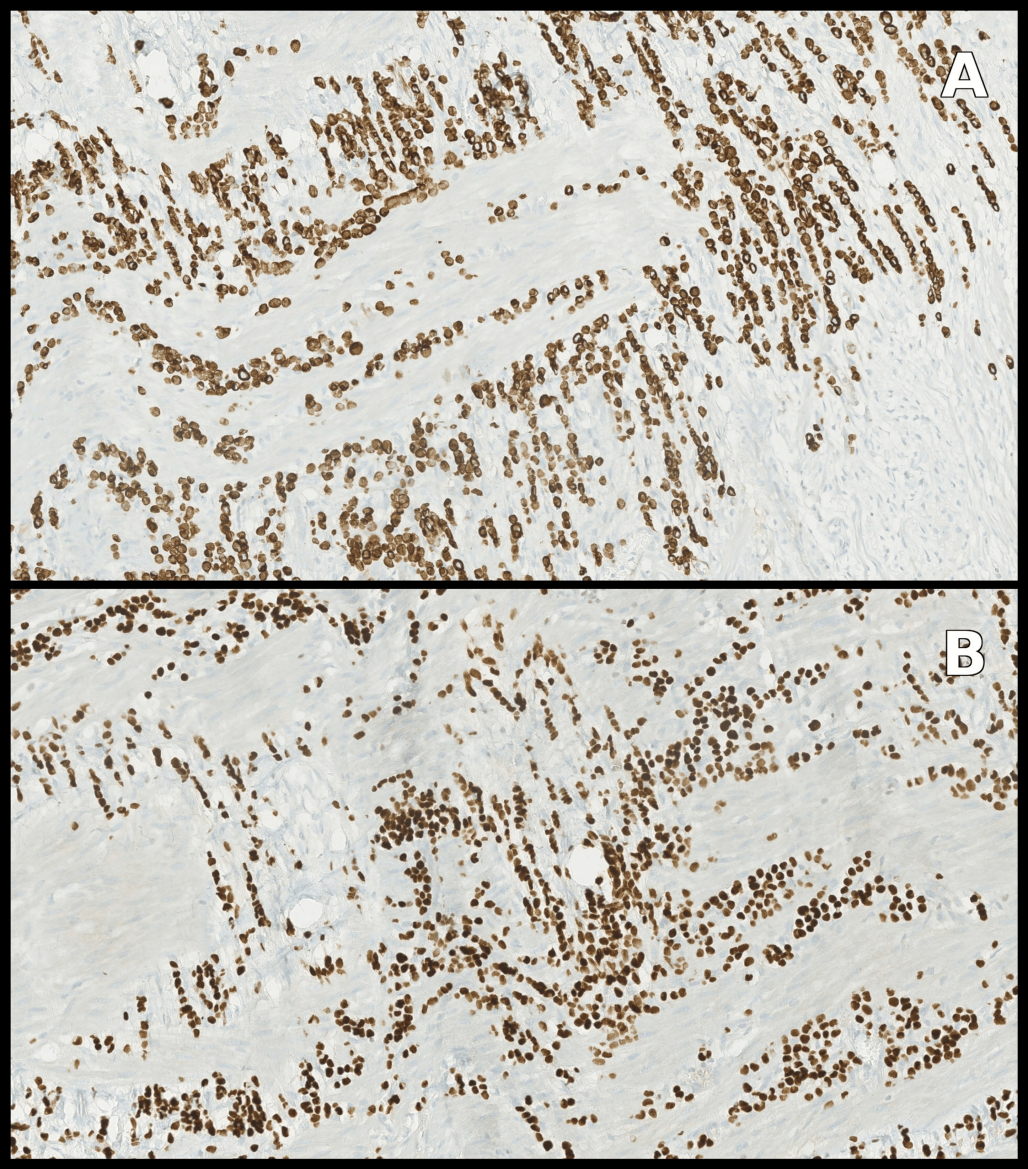
Immunohistochemistry showed strong cytokeratin 7 (A) and GATA3 expression (B).

**Figure 4:**
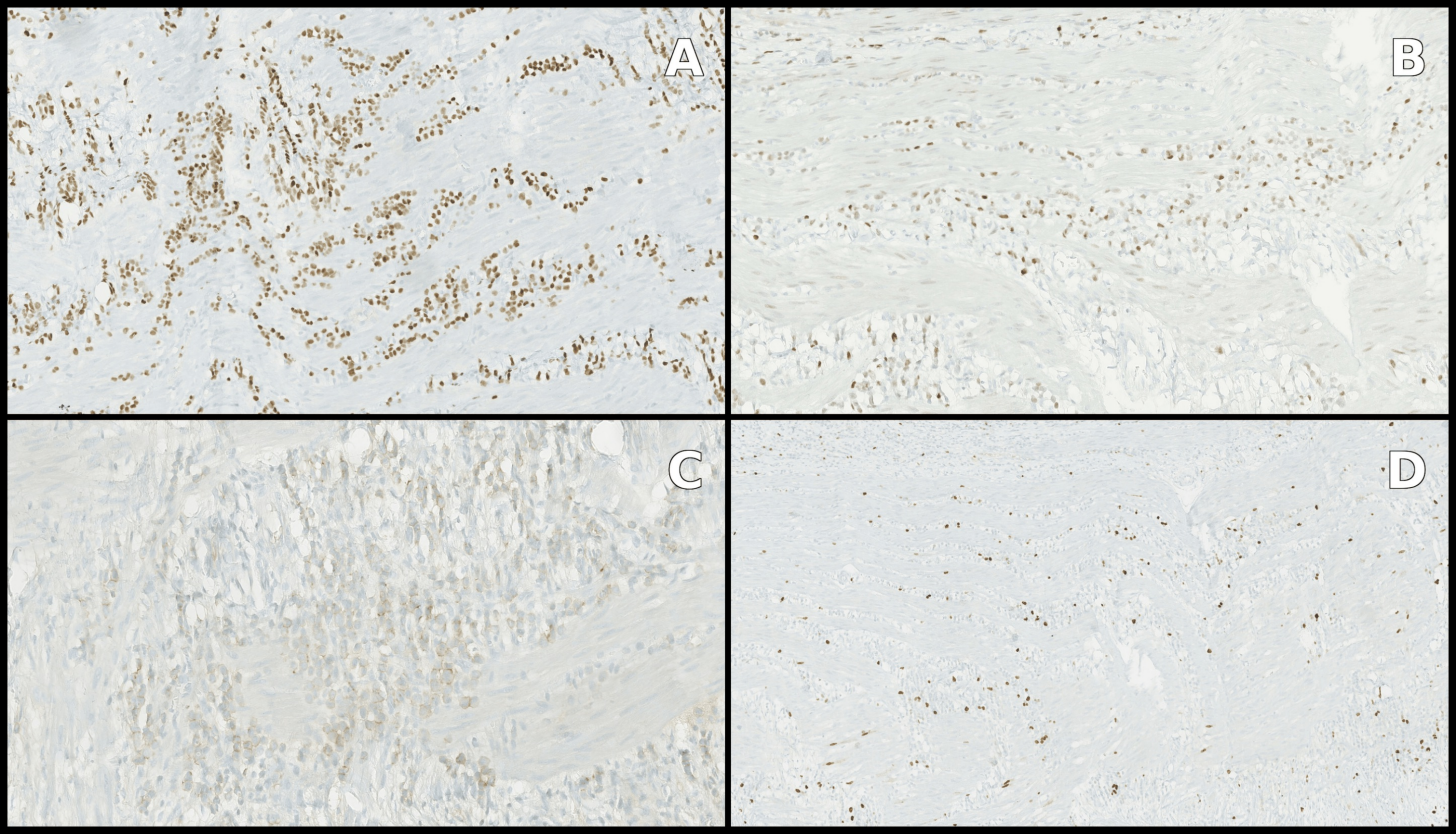
Immunohistochemistry showed strong positivity for estrogen receptors (A) and progesterone receptors (B), and low positivity for Her2 (C) and Ki-67 (D).

Histopathological analysis of the bowel resection confirmed florid peritoneal carcinomatosis due to a lobular breast carcinoma, with involvement of one metastatic lymph node. Immunohistochemistry showed strong cytokeratin 7 and GATA3 expression (Figure 3). The tumor profile was as follows: estrogen receptors: 8/8, progesterone receptors: 8/8, Her2 1+, Ki-67: 5-10% (Figure 4).

**Figure 5:**
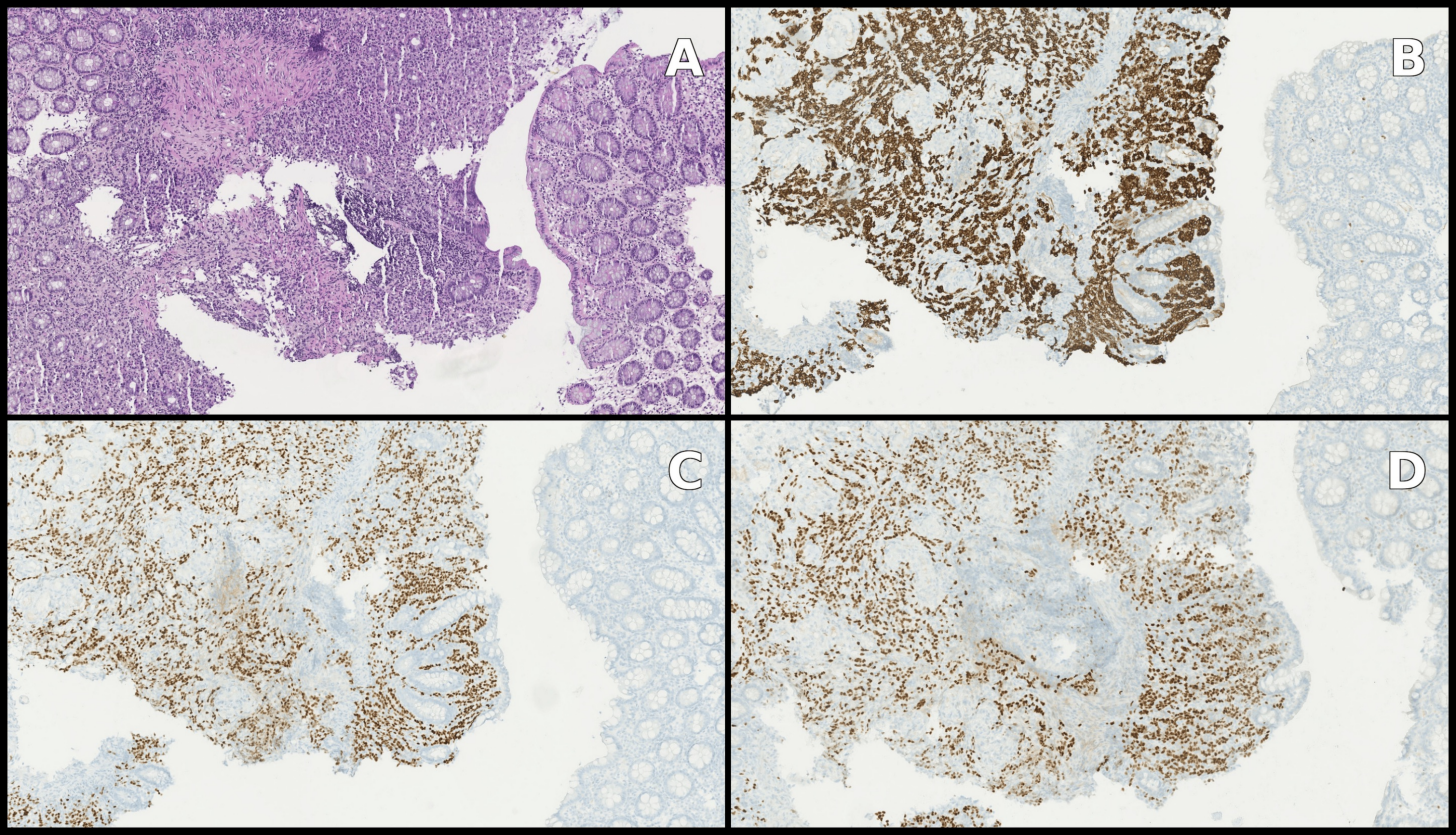
Histology and immunochemistry of the colonic polypoid lesions. Hematoxylin and eosin coloration (A), strong positivity for cytokeratin 7 (B), estrogen receptors (C), and progesterone receptors (D).

Histological examination of the colonic polypoid lesions also demonstrated submucosal infiltration by a poorly differentiated carcinoma. The immunohistochemical profile was similar to that identified in the small-bowel resection specimen, compatible with a mammary origin (Figure 5).

**Figure 6:**
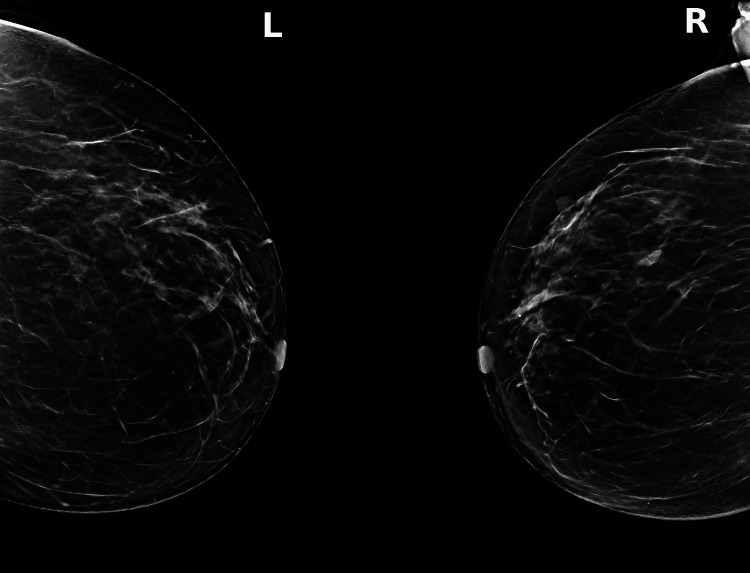
Digital mammography. Mammography yielded negative findings, revealing heterogeneously dense breast tissue (Breast Imaging Reporting and Data System (BI-RADS) C).

**Figure 7:**
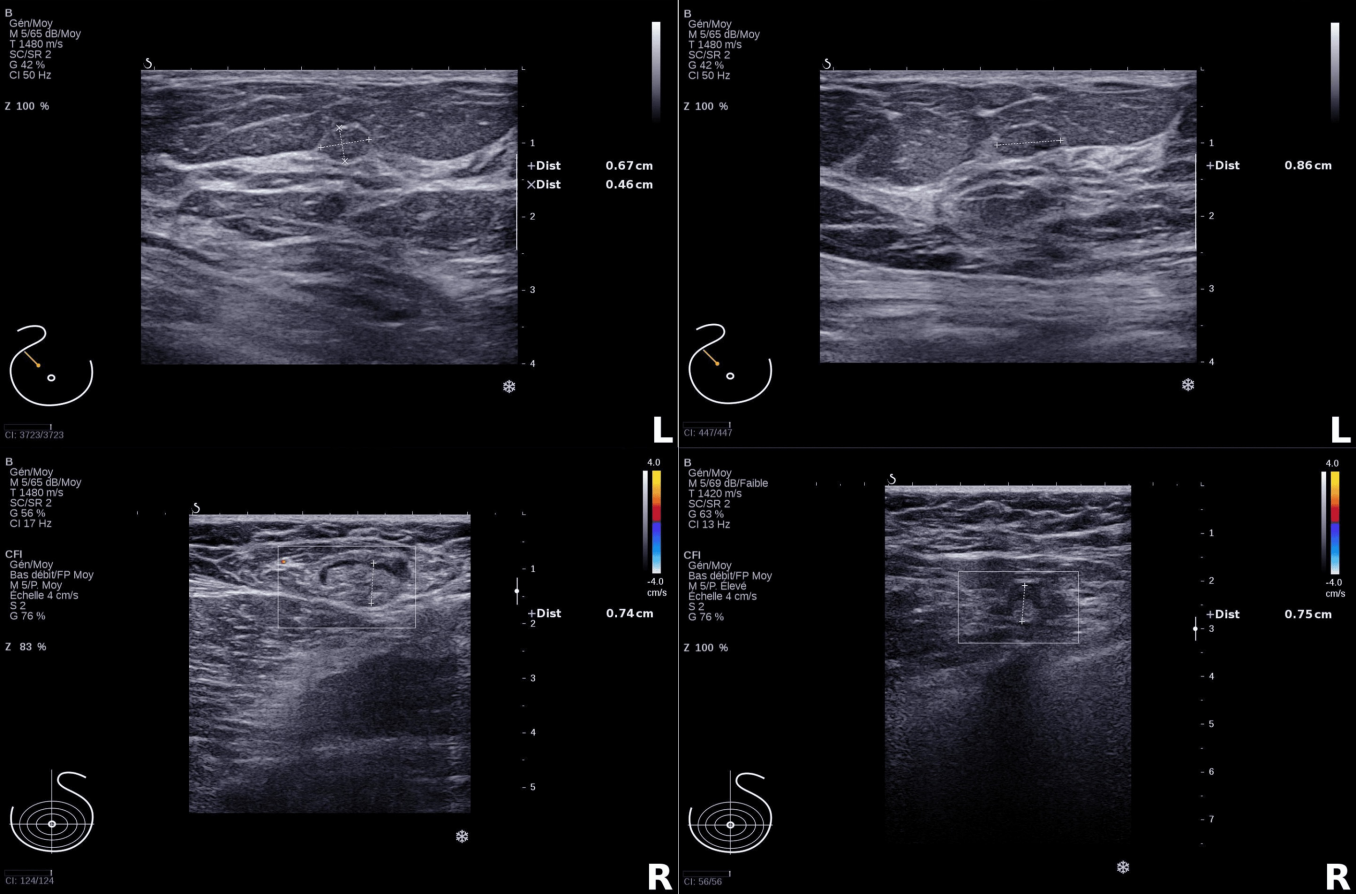
Ultrasonography of the breasts. Upper panels: transverse views of the left breast showing two well-defined, hypoechoic nodules (L). Lower panels: transverse views of the right breast showing two avascular, benign-appearing oval structures (R).

**Figure 8:**
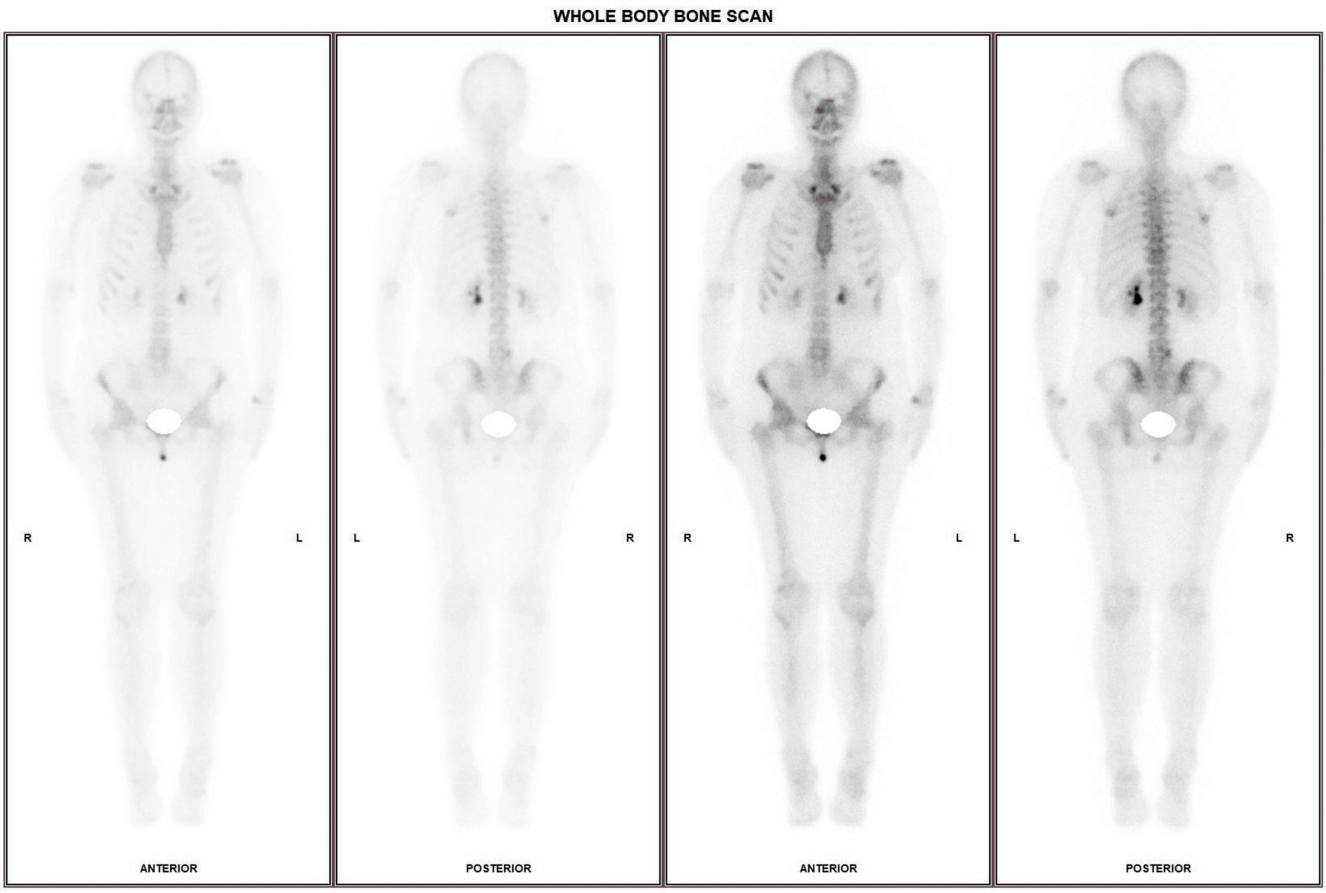
Whole-body bone scintigraphy using technetium-99m-labeled hydroxymethylene diphosphonate (99mTc-HDP). Multiple foci of moderate focal uptake in axial and appendicular joints, without evidence of metastatic involvement.

A complementary imaging workup was performed following the histopathological results. Mammography yielded negative findings, revealing heterogeneously dense breast tissue (Breast Imaging Reporting and Data System (BI-RADS) C) with a regular, oval-shaped 7 mm opacity in the outer quadrant of the right breast, while the left breast displayed a few scattered punctate microcalcifications, without any suspicious lesion (Figure 6). Ultrasound examination showed no suspicious findings. On the right, a 7 mm nodule, heterogeneous, with features consistent with a fibroadenoma, was observed. On the left, a well-circumscribed, oval-shaped, avascular lesion measuring 7 × 2 mm was identified in the supra-areolar region and was benign (Figure 7). No abnormal axillary lymph nodes were seen bilaterally. A whole-body bone scan using technetium-99m-labeled hydroxymethylene diphosphonate (99mTc-HDP) demonstrated multiple foci of moderate focal uptake, mostly in articular regions, without scintigraphic evidence of metastatic osseous involvement (Figure 8).

**Figure 9:**
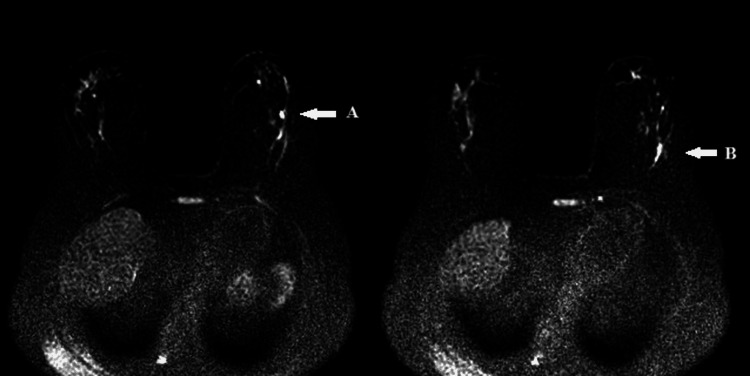
Axial diffusion-weighted breast MRI. Areas of focal enhancement in the left breast (A, B).

Breast MRI confirmed the absence of concerning findings on the right (BI-RADS 2), but revealed on the left a faintly enhancing microlobulated 20 mm lesion in the mid outer quadrant, and a 5 mm intramammary lymph node (Figure 9), consistent with a BI-RADS 4C assessment.

The postoperative course was uncomplicated. Systemic therapy was initiated shortly after surgery with endocrine-targeted treatment, namely, letrozole (aromatase inhibitor) plus ribociclib (CDK4/6 inhibitor), and has been well tolerated. At one-year follow-up, the patient remains on therapy under routine surveillance.

## Discussion

We report the case of an occult lobular breast carcinoma with negative mammography findings, revealed by an obstructive ileal metastasis. Invasive lobular carcinoma (ILC) accounts for 10-15% of breast cancers and is characterized by diffuse spread to unusual metastatic sites such as the gastrointestinal tract, ovaries, and peritoneum [3,4]. The clinical presentation in our case, displayed by an intestinal obstruction, is unusual and can, on most occasions, be mistaken for primary digestive diseases such as IBD or GI tumors, especially in emergency settings [4,5].

GI metastases of breast cancer are more frequent in ILC than in invasive ductal carcinoma (IDC), with an incidence ranging from 4% to 18% according to studies [5-7]. These metastases can occur many years after the initial breast cancer diagnosis, with reported intervals of five to 20 years [3,4]. In our case, the relatively early presentation of intestinal obstruction led to prompt diagnosis, despite the occult nature of the primary breast lesion, which unusually went undetected by mammography or ultrasound.

The diagnosis of GI metastases from ILC is often challenging due to the insidious progression of the disease. Immunohistochemical markers play a crucial role in distinguishing primary gastrointestinal tumors from metastatic breast cancer. CK7 positivity, as observed in our patient, is typical of breast cancer metastases [4-6]. The low Her2 expression, combined with strong estrogen and progesterone receptor positivity, is characteristic of ILC and oriented the treatment toward hormonal therapy [5,6].

Management of GI metastases in breast cancer requires a multidisciplinary approach. Surgery is often necessary in cases of symptomatic obstruction, like in this case, although its impact on overall survival remains limited [5,6]. In this case, segmental small bowel resection was successfully performed, followed by chemotherapy and hormone therapy. However, the prognosis remains guarded, with a median survival of 28 months reported in the literature [4,5].

## Conclusions

This case emphasizes the importance of considering metastatic breast cancer in the differential diagnosis of acute intestinal obstruction in emergency settings. Physicians, especially in the emergency department, should remain vigilant in patients presenting with atypical gastrointestinal symptoms in the presence of suggestive but inconclusive imaging findings, even in the absence of clear clinical signs of breast cancer. When mammography and ultrasound are inconclusive in suspected occult breast cancer, breast MRI plays a pivotal role in detection and staging.
